# Identifying Biomarkers from Transcriptomic Signatures in Renal Allograft Biopsies Using Deceased and Living Donors

**DOI:** 10.3389/fimmu.2021.657860

**Published:** 2021-07-01

**Authors:** Bin Yang, Nicolas Sylvius, Jinli Luo, Cheng Yang, Zhanyun Da, Charlottelrm Crotty, Michael L. Nicholson

**Affiliations:** ^1^ Department of Cardiovascular Sciences, University of Leicester, Leicester, United Kingdom; ^2^ Research and Innovation, University Hospitals of Leicester, Leicester, United Kingdom; ^3^ Nantong-Leicester Joint Institute of Kidney Science, Department of Nephrology, Affiliated Hospital of Nantong University, Nantong, China; ^4^ Genomics Core Facility, University of Leicester, Leicester, United Kingdom; ^5^ Bioinformatics and Biostatistics Support Hub Leicester, University of Leicester, Leicester, United Kingdom; ^6^ Department of Urology, Zhongshan Hospital, Fudan University, Shanghai, China; ^7^ Shanghai Key Laboratory of Organ Transplantation, Shanghai, China; ^8^ Department of Rheumatology and Immunology, Affiliated Hospital of Nantong University, Nantong, China; ^9^ Department of Surgery, Addenbrooke’s Hospital, University of Cambridge, Cambridge, United Kingdom

**Keywords:** biomarkers, differentially expressed genes, fibrosis, immunity, inflammation, transplant kidney injury

## Abstract

The survival of transplant kidneys using deceased donors (DD) is inferior to living donors (LD). In this study, we conducted a whole-transcriptome expression analysis of 24 human kidney biopsies paired at 30 minutes and 3 months post-transplantation using DD and LD. The transcriptome profile was found significantly different between two time points regardless of donor types. There were 446 differentially expressed genes (DEGs) between DD and LD at 30 minutes and 146 DEGs at 3 months, with 25 genes common to both time points. These DEGs reflected donor injury and acute immune responses associated with inflammation and cell death as early as at 30 minutes, which could be a precious window of potential intervention. DEGs at 3 months mainly represented the changes of adaptive immunity, immunosuppressive treatment, remodeling or fibrosis via different networks and signaling pathways. The expression levels of 20 highly DEGs involved in kidney diseases and 10 genes dysregulated at 30 minutes were found correlated with renal function and histology at 12 months, suggesting they could be potential biomarkers. These genes were further validated by quantitative polymerase chain reaction (qPCR) in 24 samples analysed by microarray, as well as in a validation cohort of 33 time point unpaired allograft biopsies. This analysis revealed that SERPINA3, SLPI and CBF were up-regulated at 30 minutes in DD compared to LD, while FTCD and TASPN7 were up-regulated at both time points. At 3 months, SERPINA3 was up-regulated in LD, but down-regulated in DD, with increased VCAN and TIMP1, and decreased FOS, in both donors. Taken together, divergent transcriptomic signatures between DD and LD, and changed by the time post-transplantation, might contribute to different allograft survival of two type kidney donors. Some DEGs including FTCD and TASPN7 could be novel biomarkers not only for timely diagnosis, but also for early precise genetic intervention at donor preservation, implantation and post-transplantation, in particular to effectively improve the quality and survival of DD.

## Introduction

Kidney transplantation is a life-change treatment for end-stage renal failure patients. It has been reported that 1-year allograft survival increased to around 90% in deceased donors (DD) and 95% in living donors (LD), but 10-year survival fell to 51% and 68% respectively (1, 2). Immunological and non-immunological factors affect chronic allograft injury (CAI) and allograft survival *via* different mechanistic signaling pathways (3, 4), which need to be explored. Serum creatinine (SCr) or estimated glomerular filtration rate and histopathological score have been widely used, but clinical limitations also appeared in predicting early CAI (5). Virtually no conventional methods or available biomarkers well fit clinical requirements in timely diagnosis and personalized therapy (6).

High throughput genomic technologies, such as microarray, enable investigating hundreds of thousands of genes in one sample at one time, and identifying differentially expressed genes (DEGs) involved in allograft/recipient survival. The microarray analysis has been used to disclose the mechanism of CAI (7), delayed graft function (DGF) (8), rejection (9) and the nephrotoxicity of calcineurin inhibitors (10). A meta-analysis using 150 microarray samples from ischemia-reperfusion (IR) kidneys identified DEGs, corrected the bias in models and species. 26 DEGs including LCN2, CCL2, HMOX1, ICAM1 and TIMP1 were associated with kidney transplantation injury (11). The enrichment of hypoxia and complement-and-coagulation pathways were found in DD kidneys, which might be targeted in donors to improve allograft survival (12). An additional multicenter prospective study reported that 13 genes from 159 renal biopsies at 3 months post-transplantation with stable renal function could discriminate allografts at high or low risk of CAI before irreversible histological damage occurred at 12 months (13). Candidate genes and/or their proteins such as HAVCR1 (KIM-1) and LCN2 (NGAL) in body fluids were also associated with kidney injury (14). The ultimate goal of these studies is to identify and validate DEGs as potential biomarkers to predict and diagnose CAI and improve post-transplantation care.

In this study, genomic analyses were performed in surveillance renal biopsies at 3 months post-transplantation, as well as in paired biopsies obtained at 30 minutes, using DD and LD, for two purposes. Purpose 1 was to identify DEGs between two types of donors (DD *vs* LD) or two time points (3 months *vs* 30 minutes) in the first cohort (discovery cohort) of 24 renal biopsies by microarray analysis. The hypothesis was that the difference between DD and LD in terms of survival might be ascribed to the panel of DEGs and maladjusted signaling pathways, and changed by time post-transplantation. Purpose 2 was to validate the DEGs previously identified and involved in kidney diseases and explore the correlation between their expression and functional and histological readouts at prolonged time points in 24 microarray samples and in a second cohort (validation cohort) of 33 renal allograft biopsies (unpaired time points) by reverse transcription quantitative polymerase chain reaction (qPCR). The hypothesis was that selected DEGs at the early time point might be potential biomarkers for early diagnosis and specific intervention of CAI to offering effective personalized post-transplantation care. This study revealed divergent transcriptomic signatures between DD and LD, changed by time post-transplantation, might contribute to the different survival of two type kidney allografts. Some of identified and validated DEGs, such as SERPINA3, SLPI, CBF, FTCD, TASPN7, VCAN, TIMP1 and FOS, could be novel biomarkers, which reflected initial donor injury, acute immune responses and adaptive immunity, and associated with inflammation, cell death, remodeling or potential fibrosis in transplant renal biopsies.

## Materials and Methods

### Study Design and Sample Collection

**Figure 1:**
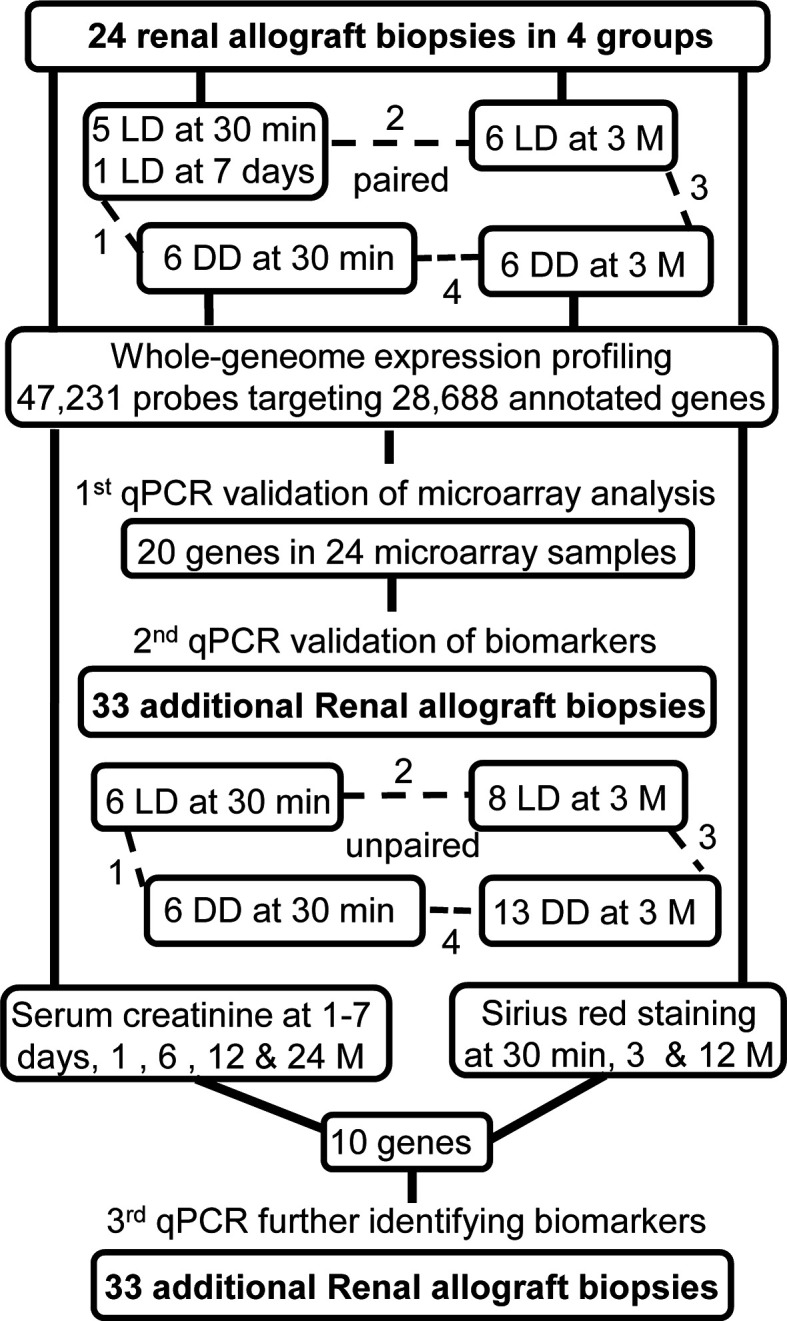
Schematic picture illustrated the study design.

In Leicester, United Kingdom, 80-100 patients per year are transplanted with kidneys: comprising 61% LD including living related and unrelated donors; and 39% DD including donation after brain death and donation after cardiac death. This study was approved by the Ethics Committee, the University Hospitals of Leicester (EDGE34225/UHL10587). From November 2008 to October 2010, 24 renal biopsies were collected from LD and DD paired at 30 minutes (a 30-minute biopsy missed replaced with one collected at day 7 of involved patient) and 3 months post-transplantation for microarray analysis, while additional 33 biopsies time point unpaired were also collected for qPCR validation (**Figure 1**). With the consent of patients, surveillance renal biopsies were obtained under ultrasound guidance. Routine biopsy samples were fixed in 10% neutral buffered formalin for histopathological diagnosis, while an additional core of renal tissue was snap-frozen in liquid nitrogen until further research analysis.

### Total RNA Extraction

Total RNAs were extracted from renal biopsies using an RNeasy^®^ Plus Mini Kit (Qiagen, West Sussex, UK) with gDNAs eliminated columns. Briefly, 10-15 mg tissues were homogenized in 350 μl of RLT Plus buffer containing guanidine thiocyanate, 1% 2-mercaptoethanol, garnet matrix and a ceramic sphere using a FastPrep^®^-24 homogenizer (MP Biomedicals, Cambridge, UK). Total RNA integrity and quantity were assessed by Bioanalyzer 2100 (Agilent Technologies, Cheshire, UK). Samples with RNA integrity number exceeding 6.5 were qualified for downstream processing (7, 15).

### Microarray Analysis

Whole-transcriptome profiling was performed using Illumina HumanHT-12 v4 Expression BeadChips (Illumina, Essex, UK), which interrogate 47,231 transcripts targeting 28,688 well-established annotated genes. Raw microarray data were normalized by quantile normalization using the control panel in the GenomeStudio Software v2010.3 (Illumina). The quality and intensity of average background were determined in the samples of compared groups. The probes with signal intensity below the average background signal of 120 were excluded.

### Reverse Transcription qPCR Validation

Total RNA was reverse-transcribed into cDNA using a Thermo Scientific RevertAid H minus First Strand cDNA Synthesis Kit (Fisher Scientific, Loughborough, UK). The primers were designed to target the same transcripts of the Illumina microarray Beadchips (**Table 1**). For each target gene, qPCR was performed using 1× Maxima SYBR Green qPCR master mix (Fisher Scientific) and 3.3 µM of forward and reverse primers. Expression values were normalized using the geometric mean of UBC, PGK1 and HPRT1, which were identified as stable housekeeping genes from microarray data. To identify potential biomarkers, 20 DEGs selected from microarray analysis, P <0.05, fold change (FC) > 1.5, and involved in renal injury, plus 2 previously interested genes CASP1 and CASP3, were further validated by qPCR in 24 microarray samples and 33 biopsies (**Supplemental Table 1**). FC > 1.5 was chosen after balancing a number of DEGs between compared groups that could best describe the nature of available data, and disclose their association with biological events referring previous publications, as well as further qPCR validation (7, 16).

**Table 1:** 20 highly DEGs selected from microarray analysis for qPCR validation.

	Name	Forward sequence	Reverse sequence
1	**SERPINA3**	CTGACCTGTCAGGGATCACA	TGCAGAAAGGAGGGTGATTT
2	**SLPI**	AATGCCTGGATCCTGTTGAC	AAAGGACCTGGACCACACAG
3	**VCAN**	CAAGCATCCTGTCTCACGAA	CAACGGAAGTCATGCTCAAA
4	**TIMP1**	CTTCTGGCATCCTGTTGTTG	AGAAGGCCGTCTGTGGGT
5	**GSTM1**	TCGTGTGGACATTTTGGAGA	GGGCTCAAATATACGGTGGA
6	**CFB**	AAGCTGACTCGGAAGGAGGT	TCCACTACTCCCCAGCTGAT
7	**FGA**	AGCCGATCATGAAGGAACAC	AAAAGCCATCCTCCCAAACT
8	**FOS**	GAGAGCTGGTAGTTAGTAGCATGTTGA	AATTCCAATAATGAACCCAATAGATTAGTTA
9	**CCND1**	CCTGTCCTACTACCGCCTCA	CCAGGTCCACCTCCTCCT
10	**DUSP1**	GTACATCAAGTCCATCTGAC	GGTTCTTCTAGGAGTAGACA
11	**CX3CL1**	TCTGCCATCTGACTGTCCTG	CTGTGCTGTCTCGTCTCCAA
12	**VHL**	AGGTCACCTTTGGCTCTTCA	TGACGATGTCCAGTCTCCTG
13	**CD14**	CGACCATGGAGCGCGCGTCCTG	GGCATGGATCTCCACCTCTA
14	**ACTRT1**	GCGTGGACTGGTAACAGGAT	TGACACAGGCAGAGGCATAG
15	**ARRDC4**	TCCCACCTGTTACTCCATCC	CCACATCTGCATAATTTGGTG
16	**TMEM149**	GAGGTGCTGGAAGAGCTGAT	CTTGCCACCACCATCTCAAT
17	**DBI**	TGGCCACTACAAACAAGCAA	TGGCACAGTAACCAAATCCA
18	**UNCSCL**	AGCTGCGGATGTTATTGGAG	TGACGGTCATGAGGTAGTGC
19	**DARC**	CTGATGGCCCTCATTAGTCC	CTCCATCTGGGAAGGAATCA
20	**SOST**	TGCTGGTACACACAGCCTTC	GTCACGTAGCGGGTGAAGTG
21	***CASP3***	AGAACTGGACTGTGGCATTGAG	GCTTGTCGGCATACTGTTTCAG
22	***CASP1***	GCTTTCTGCTCTTCCACACC	CATCTGGCTGCTCAAATGAA
23	**UBC**	ATTTGGGTCGCGGTTCTTG	TGCCTTGACATTCTCGATGGT
24	**PGK1**	AAGTGAAGCTCGGAAAGCTTCTAT	AGGGAAAAGATGCTTCTGGG
25	**HPRT1**	GCCAGACTTTGTTGGATTTGA	ATTTTGCTTTTCCAGTTTCACT

2 previously validated genes (CASP3 and CASP1) and 3 unchanged genes (UBC, PGK1 and HPRT1) were also included.

### Identifying Networks, Pathways, and Biological Functions

The Ingenuity Pathway Analysis Software v4.0 (Ingenuity^®^ Systems, Redwood City, CA) was used to map each DEG to its corresponding gene object in the Ingenuity Pathways Knowledge Base (17). P-values were calculated using a Right-Tailed Fisher’s Exact Test, which reflected the likelihood and association between a set of DEGs in the input dataset and a given process/pathway/transcription neighborhood is due to random chance. Gene networks were algorithmically generated based on their connectivity and assigned score to identify biological functions and/or diseases.

### Relevant Clinical End-Points

Clinical data including the age of donors and recipients, warm and cold ischemic time, anastomosis time, DGF and rejection were collected (18). As chosen clinical end-points SCr and Sirius red (SR) staining were also followed up either at 1-7 days, 1, 3, 6, 12 and 24 months, or 30 minutes, 3 and 12 months. SR staining representing extracellular matrix collagens I and III deposition (19–21), were performed in paraffin sections using 0.1% SR in saturated aqueous picric acid overnight. Slides were rapidly dehydrated by consequential washing in 0.01 N HCl, 70, 80, 90, 100% ethanol and xylene, and then mounted by DPX mountant. The field of entire renal cortex in each biopsy was semi-quantitatively analyzed at 400 magnification using Image Pro Software (Media Cybernetics, Bethesda, USA).

### Statistical Analysis

Non-parametric Man Whitney-U test was performed using Illumina GenomeStudio Software v2010.3. Unsupervised hierarchical clustering analysis (HCA, Manhattan average distance) and principal component analysis (PCA, autoscale) were also performed using Array Track (22). Correlation analyses between the expression level of DEGs and SCr or SR were performed with Microsoft Excel 2007 and SPSS v20 using the Pearson correlation coefficient. Clinical data such as SCr and SR staining score were expressed as means ± SEMs. Significance was assigned to P ≤ 0.05.

## Results

### Demographics of Patients and Clinical Data

Warm ischemic time was shorter, but cold ischemic time was longer in DD *vs* LD for 12 microarray analysis and 33 qPCR validation patients (**Tables 2A**, **B**). In addition, anastomosis time was also longer in DD at 30-minute qPCR validation patients. There were no significant differences in other parameters such as DGF and rejection episodes. The immunosuppression of all patients was consisted of tacrolimus, mycophenolate modetil and prednisolone.

**Table 2:** 

**(A)** Demographic and clinical characteristics of 12 patients for microarray analysis.			
**Recipient**	**LD 30 min & 3 M (n = 6)**	**DD 30 min & 3 M (n = 6)**	**P value**
Age at transplant (yr)	41.5±7.1	49.3±4.0	NS
Sex (% male)	3 (50%)	4 (67%)	
Donor age (yr)	48.7±5.6	51.7±4.7	NS
HLA mismatch	2.8±0.9	2.5±0.4	NS
Warm ischemia time (h)	0.08±0.01	0.00±0.00	P < 0.001
Cold ischemia time (h)	3.7±0.5	8.8±1.4	P < 0.01
Anastomasis time (h)	0.49±0.09	0.53±0.09	NS
Delayed graft function	0/6 (0%)	2/6 (33%)	NS
Rejection episode	2/6 (33%)	2/6 (33%)	NS
**(B)** Demographic and clinical characteristics of 33 patients for qPCR validation.		
**Recipient**	**LD 30 min (n = 6)**	**DD 30 min (n = 6)**	**P value**	**LD 3 M (n = 8)**	**DD 3 M (n = 13)**	**P value**
Age at transplant (yr)	39.5±3.2	49.7±7.0	NS	51.6±2.6	47.0±4.7	NS
Sex (% male)	5 (83%)	4 (67%)		7 (89%)	9 (69%)	
Donor age (yr)	45.3±5.6	50.7±6.9	NS	47.0±5.8	50.0±3.6	NS
HLA mismatch	2.3±0.8	3.3±1.0	NS	2.8±0.5	2.7±0.6	NS
Warm ischemia time (h)	0.08±0.01	0.02±0.02	P < 0.01	0.07±0.01	0.02 ± 0.02	P < 0.05
Cold ischemia time (h)	5.2±1.7	12.0±1.2	P < 0.01	5.1±1.3	12.5±0.9	P < 0.001
Anastomasis time (h)	0.38±0.01	0.50±0.03	P < 0.01	0.47±0.05	0.52±0.04	NS
Delayed graft function	1/6 (17%)	2/6 (33%)	NS	0/8 (0%)	2/13 (15%)	NS
Rejection episode	1/6 (17%)	2/6 (33%)	NS	0/8 (0%)	0/13 (0%)	NS

The data were expressed as means ± SEMs or % of change.

### Gene Expression Analysis

**Figure 2:**
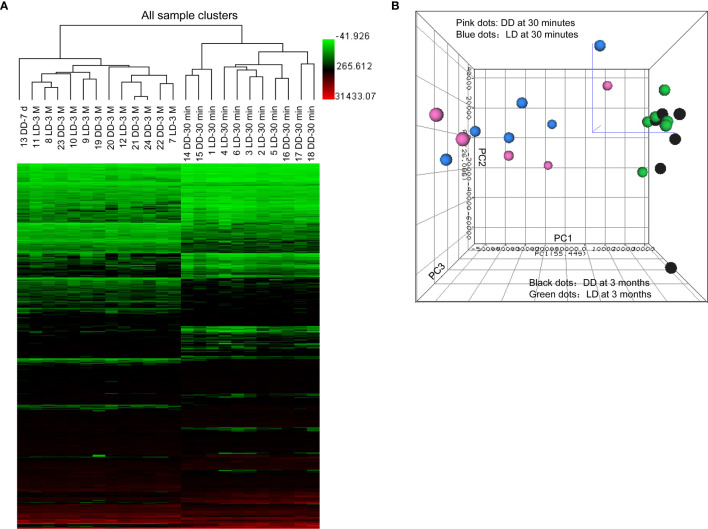
Heat map of raw data analysis of microarray, unsupervised hierarchical clustering and principal component analysis. **(A)** 899 significant DEGs (P < 0.05 and FC > 1.5) in 24 microarray analyzed samples were included in the heat map for unsupervised hierarchical clustering analysis illustrated as Euclidean + Average of non-normalized data. **(B)** Samples from biopsies at 30 minutes (dots in pink for DD and blue for LD) and 3 months (dots in black for DD and green for LD) were located into two clearly separated areas demonstrating clear differences in the overall gene profile between two different time points post-transplantation, rather than two donor types, expect one day 7 sample. The bigger was the dot size, the closer was the dot to the reader.

All confidently detected genes were analyzed by PCA and unsupervised HCA. Distinct separate clusters were revealed at 30 minutes and 3 months regardless of donor types (**Figure 2**). Interestingly, the DD biopsy collected at day 7 clearly fell into the gene cluster of 3 months.

**Figure 3:**
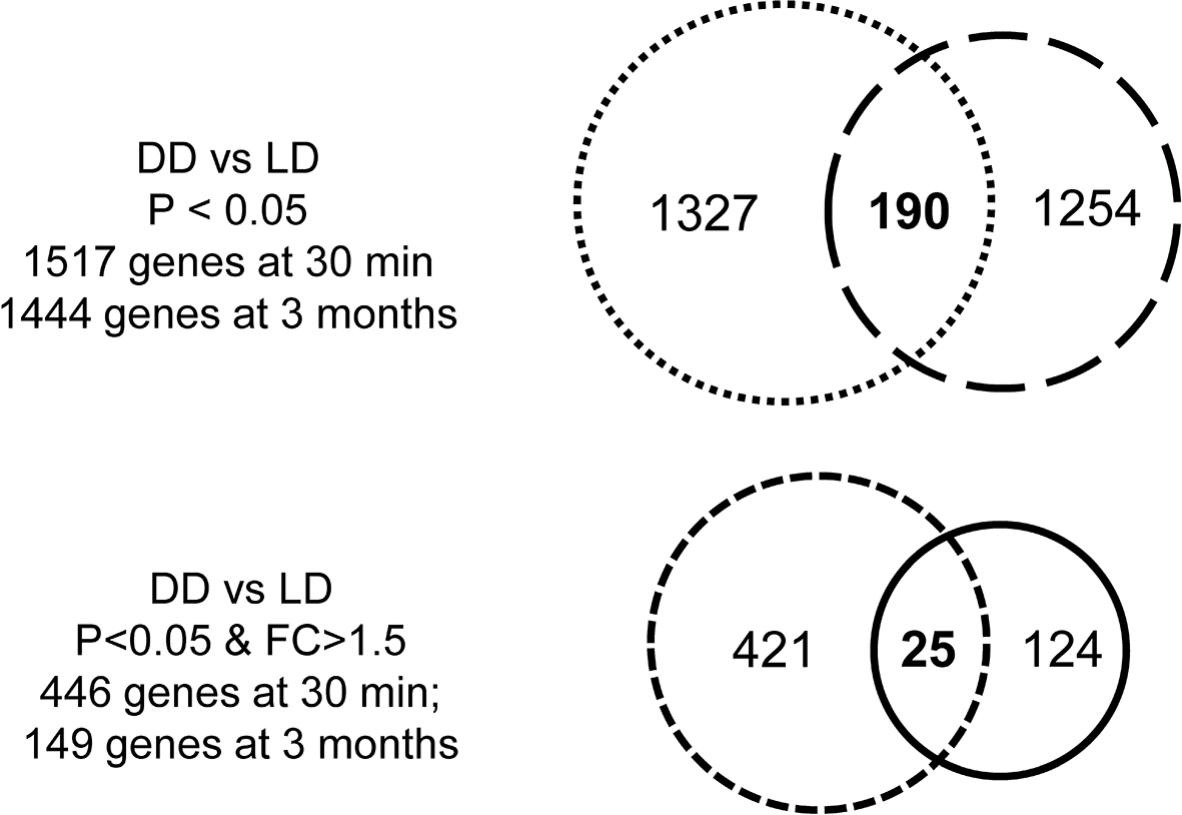
Venn diagrams of DEGs. There were 1517 DEGs (DD *vs* LD) at 30 minutes and 1444 genes at 3 months when P < 0.05, with 190 genes in common over two time point, while the number of genes was reduced 446 or 149 at each respective time points when P < 0.05 and FC > 1.5, with only 25 genes in common.

The transcriptomic profile was then assessed in 5 DD *vs* 6 LD biopsies at 30 minutes. 1735 probes corresponding to 1517 annotated DEGs (P < 0.05) were identified, with 446 DEGs at FC > 1.5 (**Figure 3**). With a view to assessing whether different DEGs were restored later, similar analyses were performed in 6 DD *vs* 6 LD biopsies at 3 months, with 11 biopsies matched to 30 minutes. 1610 probes corresponding to 1444 annotated DEGs (P < 0.05) were identified, with 149 DEGs at FC > 1.5 (**Figure 3**). The Venn diagram showed 190 commonly DEGs (DD *vs* LD, P < 0.05) between two time points, which was reduced to 25 DEGs at FC > 1.5 (**Figure 3**), including GSTM1, SOD2, CCND1 and SLPI (**Supplemental Table 2**).

**Figure 4:**
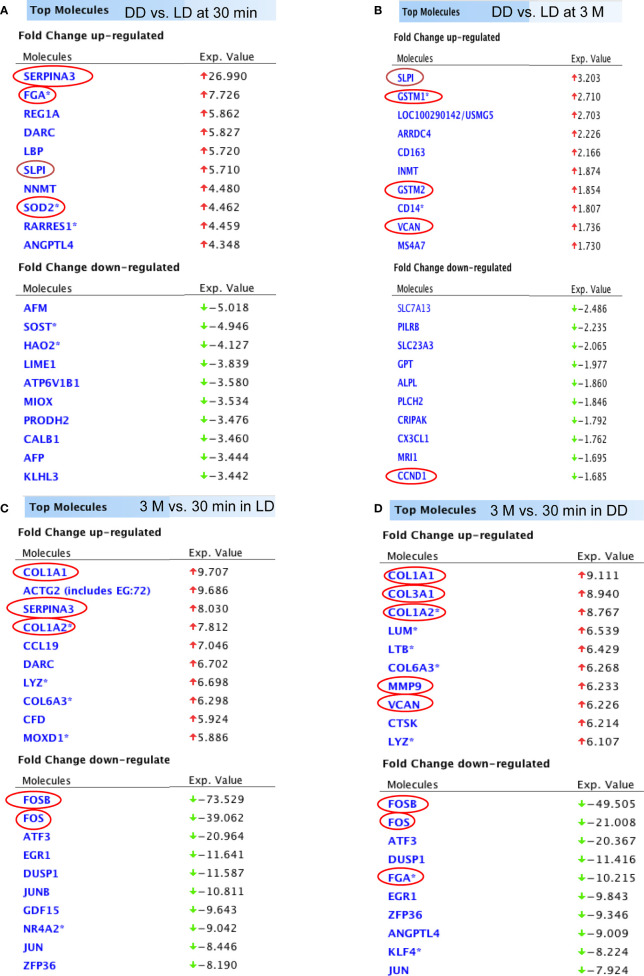
The list of DEGs. Top 10 up and down-regulated genes (DD *vs* LD, P < 0.05, FC > 1.5) at 30 minutes and 3 months were shown **(A, B)**. In addition, top 10 up and down-regulated genes (3 months *vs* 30 minutes, P < 0.05 and FC > 1.5) in LD and DD **(C, D)** respectively were also demonstrated.

The top 10 up and down-regulated DEGs (DD *vs* LD and 3 months *vs* 30 minutes) is presented (**Figure 4**). DEGs changed by time post-transplantation, with up-regulation of SERPINA3, FGA, SLPI and SOD2 at 30 minutes (27, 8, 6 and 4 fold-difference between DD *vs* LD respectively, **Figure 4A**), and 2-3 folds up-regulation of SLPI, GSTM1, GSTM2 and VCAN at 3 months in DD *vs* LD (**Figure 4B**). Interestingly, at 3 months, the two types of donors exhibited different DEGs, with up-regulation of COL3A1, MMM9 and VCAN (9, 6 and 6 fold-difference respectively) and 10-fold down-regulation of FGA in DD (**Figure 4D**). Also, at 30 minutes *vs* 3 months, SERPINA3 was found up-regulated by 8 folds in LD *vs* DD, whereas COL1A1 and COL1A2 were found upregulated by 8 to 10 folds, and FOSB, FOS, ATF3, EGR1, DUSP1, JUN and ZFP36 were downregulated by 8 to 74 folds in both donors (**Figures 4C, D**).

### Functional Annotation, Network, and Pathway Analysis

**Figure 5:**
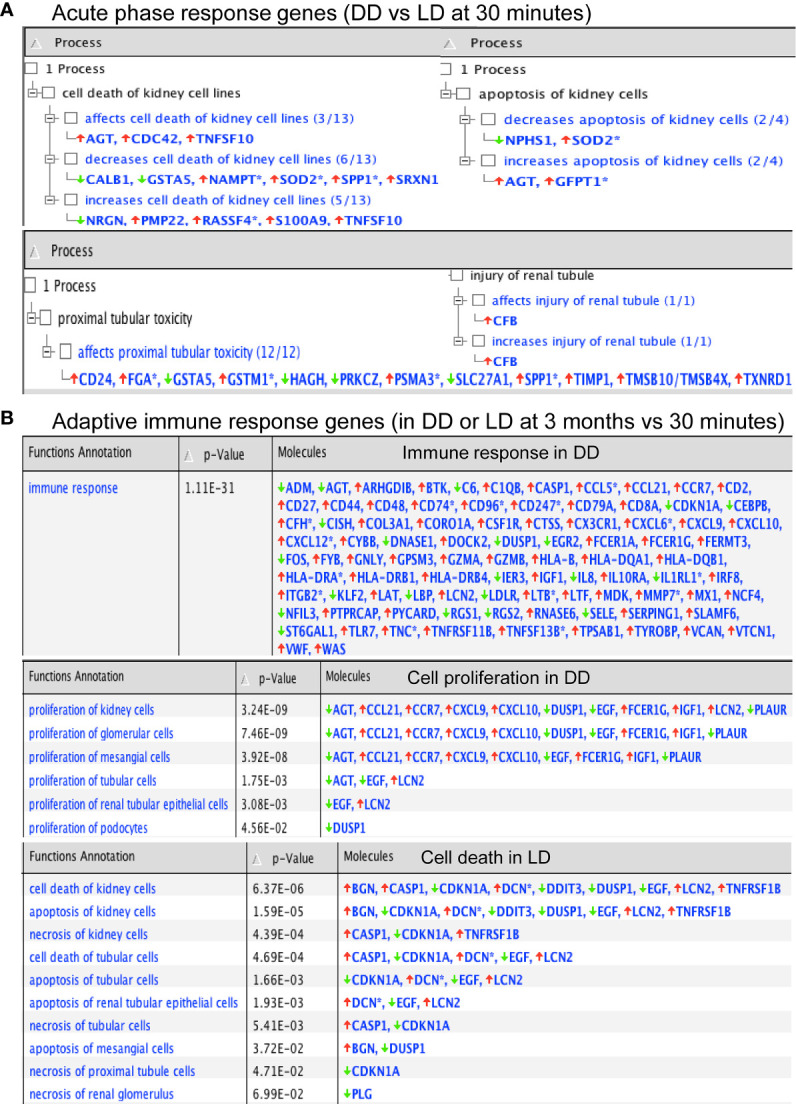
Functional annotation of DEGs in acute phase response and adaptive immunity by Ingenuity Pathway Analysis. **(A, B)** P-values were calculated using a Right-Tailed Fisher’s Exact Test to reflect the association between DEGs and a given process.

Functional annotation analysis revealed many up-regulated acute phase response genes at 30 minutes (DD *vs* LD) including AGT, CFB, TIMP1 and TNFSF10, except SERPINA3, SLPI, SOD2, GSTM1 and FGA described above, were found associated with the cell death including kidney cell apoptosis, proximal tubular toxicity and renal tubule injury (**Figure 5A**). Some of these dysregulated genes including SLPI, TIMP1 and GSTM1 also seen at 3 months are involved in oxidative stress, inflammation, tubular injury and cell proliferation (**Table 3**). In addition, adaptive immune response genes in DD or LD kidneys (3 months *vs* 30 minutes) were also revealed, for instance up-regulated CASP1, CCL5, CX3CR1, VWF, TIMP1 and LCN2 apart from COL3A1 and VCAN; and down-regulated AGT, EGF, CDKN1A and PLG apart from FGA, FOS, FOSB and DUSP1 described above, are associated with immune responses, cell death including apoptosis and necrosis, cell proliferation and tissue remodeling (**Figure 5B**).

**Table 3:** Significantly dysregulated genes in 33 additional biopsies validated by qPCR among 20 DEGs relevant to renal diseases.

Genes	Change	Comparison	P value
SERPINA3	up	CAD *vs* LD at 30 minutes	0.041^*^
SERPINA3	down	3 months *vs* 30 minutes in CAD	0.022^*^
VCAN	up	3 months *vs* 30 minutes in LD	0.028^*^
VCAN	up	3 months *vs* 30 minutes in CAD	0.081^#^
TIMP1	up	3 months *vs* 30 minutes in LD	0.031^**^
TIMP1	up	3 months *vs* 30 minutes in CAD	0.092^#^
VHL	up	3 months *vs* 30 minutes in LD	0.052^#^
CD14	up	3 months *vs* 30 minutes in LD	0.058^#^
CFB	up	3 months *vs* 30 minutes in LD	0.075^#^
UNCSCL	up	3 months *vs* 30 minutes in LD	0.079^#^
CCND1	down	CAD *vs* LD at 30 minutes	0.088^#^
UBC	down	3 months *vs* 30 minutes in LD	0.062^#^
GSTM1	down	3 months *vs* 30 minutes in LD	0.097^#^
FOS	down	3 months *vs* 30 minutes in LD	0.001^**^
FOS	down	3 months *vs* 30 minutes in CAD	0.002^**^

**P < 0.01; *P < 0.05; ^#^0.05 < P < 0.1.

**Figure 6:**
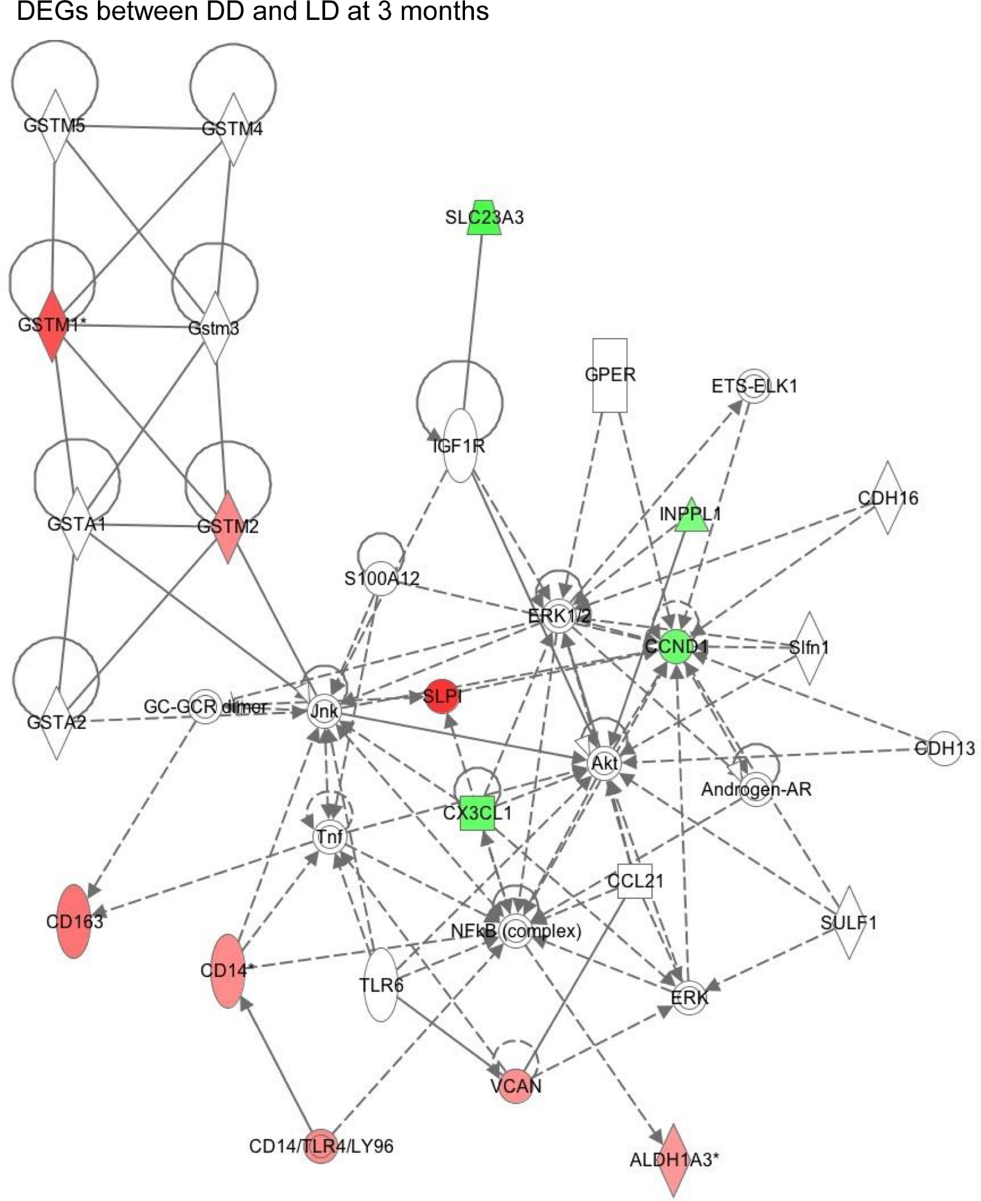
This schematic figure demonstrated an example of complicated key network links in DEGs between DD and LD at 3 months generated by function annotation using Ingenuity Pathway Analysis, as well as certain signaling pathways, with red or green color highlighted up or down-regulated genes.

**Figure 7:**
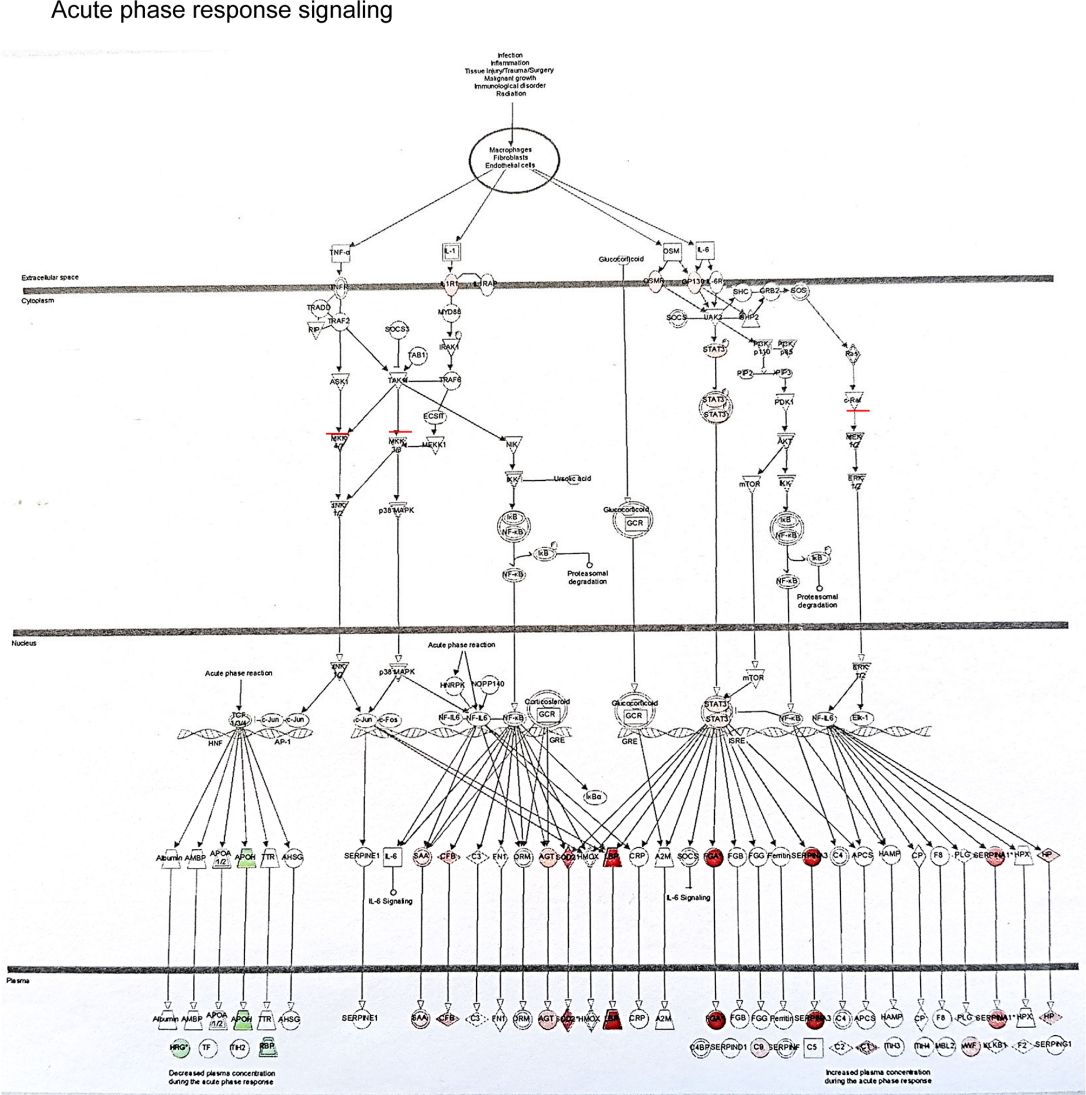
This schematic picture illustrated an example of acute phase response signaling pathways in the different compartment of cells such as the nucleus, cytoplasm and extra cellular space produced by Ingenuity Pathway Analysis, with red or green color highlighted up or down-regulated genes.

The example network of DEGs was illustrated including SLPI, GSTM1, GSTM2, CD14, CD163, VCAN and CCND1 at 3 months (DD *vs* LD, **Figure 6**), as well as the signaling pathway of GSTM1/GSTM2/SLPI-JNK-AKT-ERK-NF-ĸB-VCAN. An additional schematic picture demonstrated signaling pathways of acute phase response DEGs at 30 minutes, such as TNF-α/IL-1-JNK1/2-P38/MARK-NF-ĸB/c-FOS-SOD2/CFB, IL-6-PI3K/AKT-mTOR/STAT3-FGA/SERPINA3, and RAS-MEK1/2-ERK1/2-NF-IL6-SERPINA1 in the nucleus, cytoplasm and extracellular space including plasma (**Figure 7**).

### qPCR Validation

To validate microarray analysis, 25 genes (**Table 1**) were quantified by qPCR in the discovery cohort of samples analyzed by microarray. Excellent correlations were shown between both detection platforms with correlation coefficient R^2^ range from 0.70 to 0.94 for most DEGs, for example R^2^ = 0.974, 0.885 and 0.780 for SERPINA3, TIMP1 and CCND1 respectively at 30 minutes, R^2^ = 0.938, 0.775 and 0.832 for SLPI, VCAN, VHL respectively at 3 months, and R^2^ = 0.748 for CASP1 at both time points. The most P-values were significant, except a few genes at borderlines or not confidently detected.

These 25 genes were also quantified by qPCR in a validation cohort of 33 additional biopsies to identify potential biomarkers. In DD, up-regulated SERPINA3 and SLPI, and marginally down-regulated CCND1 were shown at 30 minutes (**Table 3**), with up-regulated TIMP1, down-regulated SERPINA3 and FOS, marginally up-regulated VCAN at 3 months. At 3 months, up-regulated TIMP1 and VCAN, and down-regulated FOS were revealed in LD, with marginally increased SLPI, CD14, CFB, VHL and UNC5CL (**Table 3**).

### Renal Function and Histological Changes

**Figure 8:**
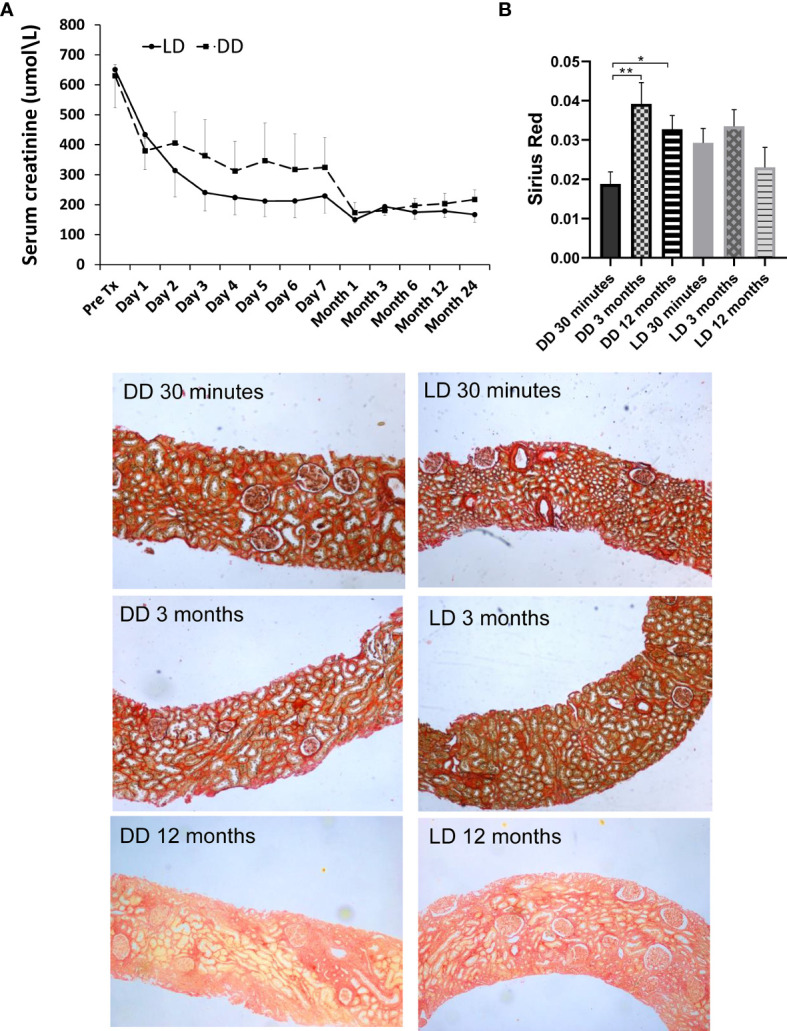
Dynamic change of renal function and Sirius Red staining in 12 patients (6 LD and 6 DD) over a prolonged period up to 2-year post-transplant. **(A)** The data of SCr were collected at pre-transplantation, day1-7 daily, 1, 3, 6, 9, 12 and 24 months post-transplantation. **(B)** The change of Sirius Red staining at 30 minutes, 3 and 12 months. The staining was mainly located in tubulointerstitial areas, which was significantly increased at 3 and 12 months compared with 30 minutes in DD, without significant difference between time points in LD. *P < 0.05; **P < 0.01.

The patients enrolled in microarray-based gene expression analysis were followed up for 24 months, with relatively stable renal function (**Figure 8A**), no graft loss, although one patient who had received a DD kidney died due to sepsis-cardiac arrest. No significant differences in SCr (obtained from the clinical database of the University Hospitals of Leicester NHS Trust) between two types of donors were shown at any time points, with a consistent better trend in LD (*vs* DD) at 2-7 days, 1, 6, 12 and 24 months, except pre-transplantation, 1 day and 3 months. Renal fibrosis assessed by SR staining in 24 microarray and additional 33 biopsies, mainly located in tubulointerstitial areas and scattered in glomerular areas, was increased in DD at 3 and 12 months compared with 30 min, without significant difference in LD (**Figure 8B**).

### Correlation Between DEGs and Clinical Outcomes

**Figure 9:**
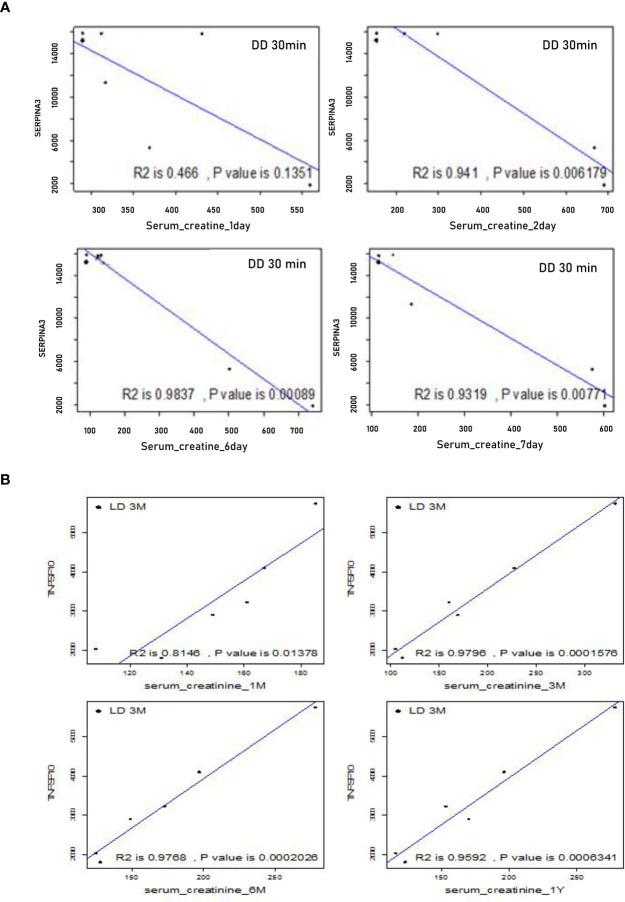
Correlations between microarray analysis detected DEGs and renal function in the first cohort of 24 biopsies. **(A)** Negative correlations between SERPINA 3 and SCr at early time points from day 1 to 7; **(B)** Positive correlations between TNFSF10 and SCr at later time points from 1, 3, 6 to 12 months.

To assess whether DEGs at 30 minutes or 3 months could be potential biomarkers to predict allograft survival, the correlations between the expression level of these DEGs, SCr and SR staining were analyzed at the same and extended time points. Using the microarray signal intensity of DD and LD at 30 minutes, a list of top 120 genes was identified significantly correlated with SCr and/or SR staining. For instance, SERPINA3 was found negatively correlated with SCr at 1-7 days, and TNFSF10 was found positively correlated with SCr at 1, 3, 6 and 12 months (**Figures 9A, B**). The expression levels of 10 DEGs (highly expressed and involved in renal physiopathology based on GO terms or previous publications) GSTM1, COQ2, CCND1, CFB, FTCD, UNC5CL, SERPINA3, RAI14, TSPAN7 and SOD2 were correlated with both SCr and SR staining at 12 months (**Supplemental Table 2**). Moreover, at 3 months TSPAN7, BTG3 or COQ2 expression was correlated with SCr, while FAU, UMOD, TSPO, IMPDH2, ADSS, RAF1, ARG2, AGTR1 or PDE6D expression was correlated with SR staining. In addition, other DEGs PTPN6, CNNM3, CSTF3, CHURC1, UCRC, COX7B, FXYD5, CD74, MRPL42P5, SENP2, TMEM129, EIF2AK1, FAM165B, C6ORF66 and ATP5J were also found in four patients with a rejection compared to those without.

Above 10 DEGs correlated with both SCr and SR staining were further validated by qPCR in the second cohort of 33 biopsies. In DD (*vs* LD), there were four up-regulated genes FTCD, SERPINA3, TASPN7 and CFB (P < 0.05) at 30 minutes, with a trend of increased SOD2 and decreased CCND1, while raised FTCD and TSPAN7 (P < 0.001) were also seen at 3 months, with marginally up-regulated CCND1 and SOD2.

**Figure 10:**
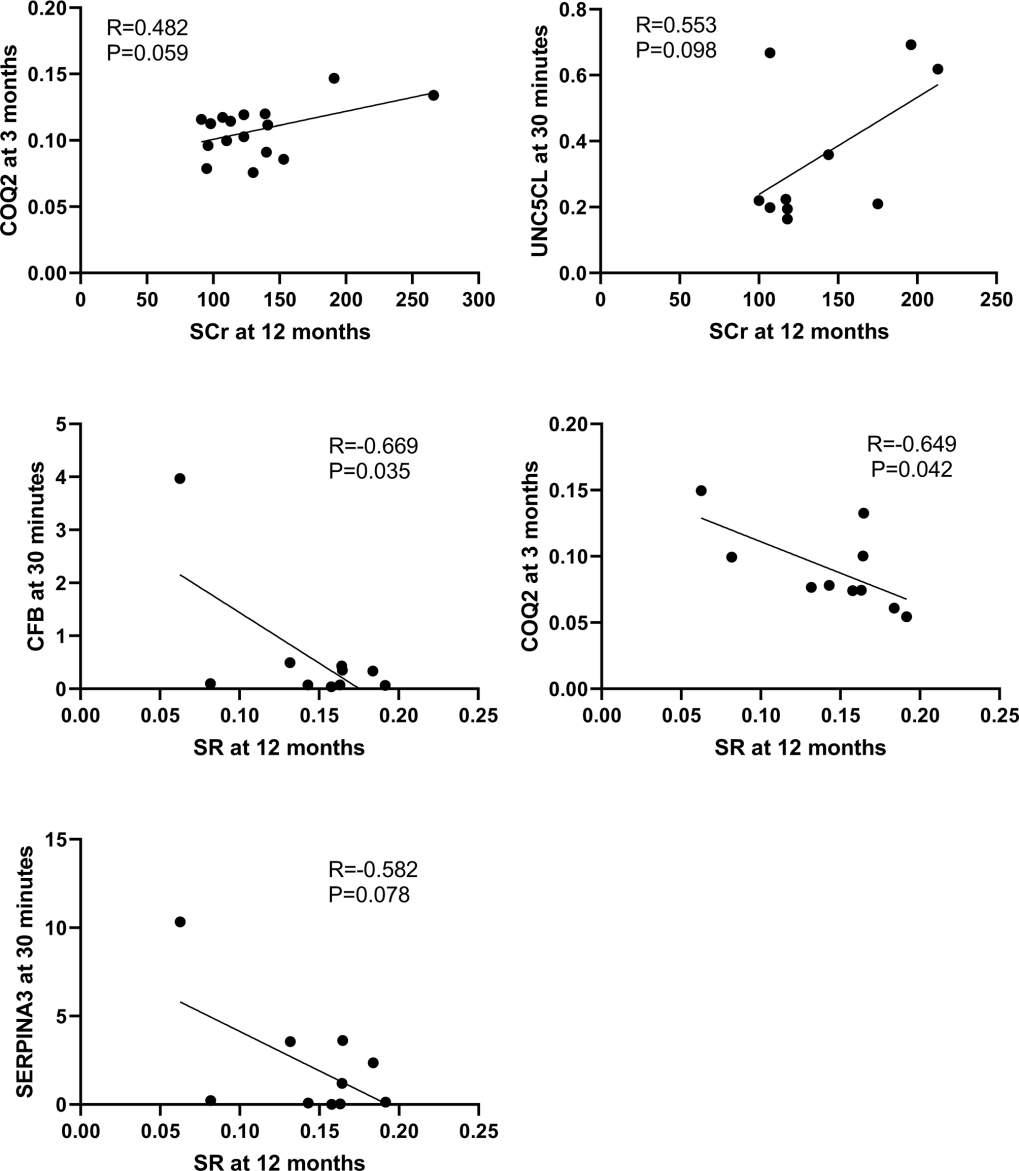
Correlations between qPCR validated DEGs at 30 minutes or 3 months and SCr or SR at 12 months in the second cohort of 33 renal biopsies. The significant positive or marginal negative correlations were shown between COQ at 3 months or UNC5CL at 3 minutes and SCr at 12 months, as well as COQ, CFB or SERPINA3 at 30 minutes and SR at 12 months, respectively.

The correlation between the 10 DEGs and SCr or SR at 12 months in the second cohort of 33 biopsies was further analyzed respectively using Pearson correlation coefficient. CFB (R=-0.669, P=0.034) and COQ2 (R=-0.649, P=0.042) at 30 minutes were significantly correlated with SR at 12 months, while UNC5CL (R=0.553, P=0.098) at 30 minutes was marginally correlated with SCr at 12 months. Other genes at 3 months such as COQ2 (R=0.482, P=0.059) or SERPINA3 (R=0.405, P=0.095) were also marginally correlated with SCr or SR at 12 months respectively (**Figure 10**).

In addition, some DEGs such as SERPINA3, SLPI, VCAN, FOS and SOD2 revealed in this study by microarray analysis, validated by qPCR and associated with kidney injury, were also reported by a previous publication (14) (**Supplemental Table 3**).

## Discussion

An increasingly severe shortage of kidney donors leads to the expansion of donor pools by including DD for transplantation. However, the survival of transplant kidneys using DD is not as good as LD. In this study, the microarray analysis of renal allograft biopsies showed divergent transcriptomic signatures between LD and DD, which shifted from acute immune responses at 30 minutes to tissue injury/repair and remodeling/fibrosis at 3 months and might contribute to different long-term survival. DEGs might be attributed to initial donor conditions, innate and adaptive immune responses, persistent inflammation associated with immunosuppressants. Some DEGs including SERPINA3, SLPI, CBF, FTCD, TASPN7, VCAN, TIMP1 and FOS might be novel biomarkers to facilitate timely diagnosis and early therapeutic intervention in donor kidney preservation, implantation or post-transplantation, in particular, effectively improve the donor quality and allograft survival of DD.

Clearly different gene clusters revealed in allograft biopsies between two time points regardless of donor types indicates time post-transplantation playing crucial roles. DEGs affected by time post-transplantation might mask initial differences of cold or warm ischemic time in donors, as donation trauma and acute immune responses were overwhelming in implantation. The dramatic changes were eased off, which was reflected by 3 times more DEGs at 30 minutes than 3 months.

We then prioritized to analyze how DEGs (DD *vs* LD) associated with allograft survival. The functional annotation revealed up-regulated acute response genes SERPINA3, FGA and SLPI, together with inflammation and nephrotoxicity associated genes SOD2, GSTM1, VCAN and TIMP1, but down-regulated repair related genes FGA, CCND1 and FOS in DD at 30 minutes. More fibrotic genes COL3A1, TIMP4 and MMP9 were raised in DD, with increased TIMP1, VCAN, COL1A1 and COL1A2 in both donors at 3 months. The dynamic change of these DEGs well reflected donor initial injury and recipient adaptive immunity *via* different networks and signaling pathways such as TNF-α/IL-1-JNK1/2-P38/MARK-NF-ĸB/c-FOS-SOD2/CFB and GSTM1/GSTM2/SLPI-JNK-AKT-ERK-NF-ĸB-VCAN. These results were consistent with a study using 59 protocol kidney biopsies that showed immune pathway activation, fibrotic gene expression and cell proliferation-repair-remodeling at 1, 3 and 12 months respectively (7). 40% DEGs and 50% pathways initially activated were persisted to 3 months, while pro-fibrotic genes were expressed before observed microscopic interstitial fibrosis, suggesting that DEGs might be early biomarkers.

FGA protein is crucial in coagulation, inflammation and tissue regeneration. Soluble fibrinogen-like protein 2 (sFGL2) increased in the circulation of allograft rejection patients, contributed to the apoptosis of cultured tubular epithelial cells (TECs), which is detrimental in early injury, but also initiates remodeling (23, 24). Increased sFGL2 in the recovery stage of auto-transplanted porcine kidneys was associated with inflammatory cell apoptosis and decreased inflammation (25). However, reduced FGA in the plasma of FGA^+/-^ mice protected IR kidneys against TEC death and inflammation, with increased CCND1 and proliferation (26). Taken together, down-regulated FGA and CCND1 in DD at 3 months might affect allograft recovery.

SOD2 encoding mitochondrial enzyme protects against IR injury and inflammation (27). SOD2 was found decreased in the urine of aged-mice with increased oxidative stress, apoptosis tubulointerstitial fibrosis and proteinuria (28). GSTM1 protects against xenobiotic compounds and toxicity caused by immunosuppressants in renal transplant recipients. Up-regulated GSTM1 was protective to increased oxidative stress in chronic kidney disease (29). However, GSTM1 was also linked to high rejection risks and unfavorable to long-term allograft outcomes (30, 31). In this study, highly expressed SOD2 and GSTM1 in DD at 30 minutes and 3 months might reflect initial donor injury, as well as self-defense.

Strong positive correlations between two detection methods were shown for most detected DEGs, and potential biomarkers were also identified and validated by qPCR. Up-regulated SERPINA3 and SLPI were shown in DD at 30 minutes with SLPI remained high at 3 months, and raised both in LD at 3 months. SERPINA3 is a secreted acute phase protein associated with inflammatory diseases, a potential pharmacological target (32, 33). Elevated serum SERPINA3 in mice increased the transendothelial permeability of retina associated with diabetic retinopathy (34). Urine SERPINA3 was positively correlated with the activity of lupus nephritis, with SERPINA3 located in endothelial cells and TECs (35), and predicted renal inflammation and fibrosis, especially early transition of AKI to CKD (36). SLPI protein, an inhibiting proteolytic enzyme, participates in mucosa anti-microbial defense by mediating the production of anti-inflammatory cytokines, IL-10 and TGF-β (37). Up-regulated SLPI in epithelial cells plays active roles in defending airways upon inflammation (38). SLPI expressed in TECs of heathy renal biopsies and elevated in serum of uremic patients (39) to regulate proteolytic activity in inflammatory sites (40). SLPI in plasma and urine were increased in AKI post-transplantation, aortic aneurysm repair and cardiac surgery (41–43). SERPINA3 and SLPI, therefore, might be ideal biomarkers of kidney injury.

TIMP1 and VCAN were up-regulated in both DD and LD at 3 months, with reduced FOS. TIMP1 associated with renal IR injury (11), together with matrix metalloproteinases (MMPs), plays important roles in the progression of CAI (44). Urinary/serum TIMP1/MMPs was/were active in acute tubulointerstitial injury/inflammation (45). VCAN, an indicator of AKI post-transplantation and ongoing parenchymal injury, predicts allograft loss (14). FOS, a transcription factor, involves in MAPK signaling pathway (46). FOS protein has been described as stimulating central opioid receptors, activated renal sympathetic nerves and enhanced IR-induced AKI in mice (47). c-FOS and VEGF play synergistic roles in inflammation and angiogenesis in the peritoneal membrane upon inflammation and lead to ultrafiltration failure (48). The selective inhibitor of c-FOS/activator protein-1 (AP-1, a redox-sensitive transcription factor) inhibited proinflammatory cytokines and improved the survival of lipopolysaccharide-induced AKI (49).

Of note, in the top 10 down-regulated gene list, 7 genes FOSB, FOS, ATF3, EGR1, DUSP1, JUN and ZFP36 were down-regulated in both LD and DD at 3 months. All of them are transcription factors, and immediate early genes in the mouse mononuclear phagocyte system, while they co-expressed with cytokines and chemokines indicates disaggregated cells (50). Hydrogen peroxide-induced apoptosis in mesangial cells *via* JNK/c-FOS/c-JUN/AP-1 pathway (51). ATF3, a rapidly induced transcription factor by IR, strongly represses the transcription of inflammatory cytokines, plays essential roles in anti-apoptosis, anti-migration and anti-inflammation (52). EGR-1 is urea-inducible early gene transcription factor in renal inner medullary collecting ducts (53). Biological effects of these genes on CAI are worthy to explore.

Finally, the expression level of 10 DEGs at 30 minutes were found positively corrected with both renal function and histology at 12 months and were validated in 33 additional biopsies to identify more biomarkers. Up-regulated SERPINA3, FTCD, TSPAN7 and CFB were confirmed in both DD and LD at 30 minutes. FTCD, a liver-specific enzyme integrating Golgi complex with vimentin filament cytoskeleton, is linked to autoimmune hepatitis and glutamate formiminotransferase deficiency (54, 55). FTCD appears to be the molecular “glue” crosslinking vimentin filaments into fibers (56). TSPAN7 plays a role in cell and membrane compartmentalization and regulates the trafficking and function of its partner proteins (57), associated with cancer metastasis-suppressive interactions (58–60). It has been hard to find a direct link between FTCD or TSPAN7 and kidney diseases so far. CFB is involved in the complement alternative pathway and atypical hemolytic uremic syndrome (aHUS) (61). Recurrence risk and kidney allograft outcome in recipients with aHUS were associated with thrombotic microangiopathy and *de novo* CFB mutation (62, 63). CBF was highly expressed in DD at 30 minutes and fell at 3 months, but increased in LD, implying that CBF, similar to SERPINA3, might be adjusted at different stages to limit damage and encourage remodeling.

Nevertheless, there are certain limitations in this study. A small size of 24 biopsies were used for microarray analysis, but DEGs were revealed between DD and LD at as early as 30 minutes after implantation (a precious therapeutic window), and some of these DEGs were persistent at 3 month. In addition, selected DEGs were also further validated by qPCR in the 24 microarray analyzed samples and additional 33 time point unpaired renal biopsies, and were correlated with renal function and renal fibrosis at extended time points up to 24 months. CASP1 and CASP3, intensively investigated in our previous studies, were not on the list of DEGs, but their importance in transplant-related injuries cannot be excluded as FC > 1.5 and P < 0.05 were selected.

The preliminary data from this study need to be cross-validated in large clinic cohorts with long-term follow-up, or in subgroups with/without DGF and/or rejection. There was no clinical difference in terms of DGF and rejection between LD and DD, but DEGs such as PTPN6, CNNM3, CSTF3, CHURC1 and UCRC were revealed in four patients with a rejection compared to without rejection. To validate these DEGs as biomarkers, analyzing samples collected from a large cohort of patients who received either LD or DD kidneys and showed clear allograft dysfunction, rejection or loss would be ideal dataset to explore associations between DEGs measured before 3 months post-transplantation and adverse outcomes or long-term survival. It has been reported that other DEGs detected by microarray such as HuMig and MIP-3β were abundant in patients with acute rejection (64), CD20 was associated with B cell infiltration and acute rejection (9), and KRT15 and HOXB7 at 6 months were linked to chronic rejection at 12 months (**Supplemental Table 3**) (65).

The microarray analysis used cDNA synthesized based on total RNA extracted from kidney homogenates might average DEGs in different cells/cell types due to their heterogeneity in kidneys (66). Laser captured microdissection and single cell sequence may be solutions. The relations between DEGs, upstream regulating miRNAs, corresponding proteins and downstream biological events were difficult to fully dissect. Current translational human studies require concurrent genomic, proteomic and metabolomic analysis in small tissues (67). The real time central molecular assessment of kidney transplant biopsies based on Molecular Microscope Diagnostic System classifier algorithms offers a useful new dimension in biopsy interpretation (68), although mining DEGs in bioinformatics data and transferring to clinical applications are still challenging.

Moreover, a great potential of gene therapy using RNAi has been demonstrated in translational medicine. Registered clinical trials of siRNAs (www.ClinicalTrials.gov) rapidly increased, only I5NP targeting p53 has been validated in kidney injury. siRNA target caspase-3 was renoprotective in our previous studies used a serial of biological models including TECs and mouse IR kidneys (69, 70). In particular, local and systemic administrating serum stabilized caspase-3 siRNA effectively silenced caspase-3, favorably changed serum cytokines, reduced apoptosis and inflammation, and protected cold static or normothermic machine preserved and auto-transplant porcine kidneys (71, 72). Therefore, siRNA therapy is promising in preservation/resuscitation donors and reducing declined rate of DD (73), and in implantation and post-transplantation to effectively prolong allograft survival.

In conclusion, the transcriptional profile of allograft biopsies is different between 30 minutes and 3 months, with more DEGs between DD and LD at 30 minutes reflected donor injury and recipient innate immunity. Consistent DEGs at 3 months mainly represented adaptive immunity, remodeling or fibrosis. Some DEGs such as SERPINA3, SLPI, CBF, FTCD, TSPAN7, VCAN, TIMP1 and FOS might be novel biomarkers for not only timely diagnosis, but also facilitating precise genetic intervention in donor preservation, implantation and the early stage of post-transplantation, monitoring CAI progression and therapeutic responses, and effectively improving the donor quality and allograft survival of DD.

## Data Availability Statement

The datasets presented in this study can be found in an online repository of Gene Expression Omnibus (GEO): https://www.ncbi.nlm.nih.gov/geo, and the accession number of the GEO: GSE178689.

## Ethics Statement

The studies involving human participants were reviewed and approved by the Ethics Committee, the University Hospitals of Leicester (EDGE34225/UHL10587). The patients/participants provided their written informed consent to participate in this study.

## Author Contributions

BY, NS, and MN conceived and designed the study. BY, NS, CY, ZD, JL, and CC acquired, analyzed, and interpreted the data. BY and NS wrote the paper. BY approved the final submission of the manuscript. All authors contributed to the article and approved the submitted version.

## Funding

This study was supported the UK-China Fellowship for Excellence, Department for Business Innovation and Skills (2010/2011) and the National Nature Science Foundation of China (81170689, 81570677 and 81873622).

## Conflict of Interest

The authors declare that the research was conducted in the absence of any commercial or financial relationships that could be construed as a potential conflict of interest.

## Supplementary Material

Supplementary Table 1 Details of 20 selected DEGs (P < 0.05, FC > 1.5) relevant to kidney diseases. FC, fold change. Grey color highlighted DEGs either commonly expressed in two time points or correlated with both SCr and SR staining.

Supplementary Table 2 25 DEGs (No.1-25) between DD & LD in common at 30 min and 3 months, and 10 DEGs (No.2, 3, 19 and 26-32) at 30 minutes correlated with both SCr and SR staining for further qPCR detection to identify biomarkers. SCr, serum creatinine; SR, Sirius red staining.

Supplementary Table 3 DEGs revealed by microarray analysis and associated with kidney injury or allograft rejection from this study and previous publications. There were some commonly DEGs (underline) between studies.

## References

[1] Ibrahim M Vece G Mehew J Johnson R Forsythe J Klassen D. An International Comparison of Deceased Donor Kidney Utilization: What can the United States and the United Kingdom Learn From Each Other? Am J Transplant (2020) 20:1309–22. 10.1111/ajt.15719 31758833

[2] Cecka JM. The UNOS Renal Transplant Registry. Clin Transplants (2002), 1–20. 12971433

[3] Akalin E O’Connell PJ. Genomics of Chronic Allograft Injury. Kidney Int Suppl (2010) 119:S33–7. 10.1038/ki.2010.420 21116315

[4] Kezic A Stajic N Thaiss F. Innate Immune Response in Kidney Ischemia/Reperfusion Injury: Potential Target for Therapy. J Immunol Res (2017) 2017:6305439. 10.1155/2017/6305439 28676864 PMC5476886

[5] Yilmaz S Isik I Afrouzian M Monroy M Sar A Benediktsson H. Evaluating the Accuracy of Functional Biomarkers for Detecting Histological Changes in Chronic Allograft Nephropathy. Transplant Int: Off J Eur Soc Organ Transplant (2007) 20:608–15. 10.1111/j.1432-2277.2007.00494.x 17521383

[6] Muhlberger I Perco P Fechete R Mayer B Oberbauer R. Biomarkers in Renal Transplantation Ischemia Reperfusion Injury. Transplantation (2009) 88:S14–9. 10.1097/TP.0b013e3181af65b5 19667956

[7] Vitalone MJ O’Connell PJ Wavamunno M Fung CL Chapman JR Nankivell BJ. Transcriptome Changes of Chronic Tubulointerstitial Damage in Early Kidney Transplantation. Transplantation (2010) 89:537–47. 10.1097/TP.0b013e3181ca7389 20147884

[8] Guerrieri D Re L Petroni J Ambrosi N Pilotti RE Chuluyan HE. Gene Expression Profile in Delay Graft Function: Inflammatory Markers are Associated With Recipient and Donor Risk Factors. Mediators Inflamm (2014) 2014:167361. 10.1155/2014/167361 24959002 PMC4052172

[9] Sarwal M Chua MS Kambham N Hsieh SC Satterwhite T Masek M. Molecular Heterogeneity in Acute Renal Allograft Rejection Identified by DNA Microarray Profiling. N Engl J Med (2003) 349:125–38. 10.1056/NEJMoa035588 12853585

[10] Vitalone MJ Ganguly B Hsieh S Latek R Kulbokas EJ Townsend R. Transcriptional Profiling of Belatacept and Calcineurin Inhibitor Therapy in Renal Allograft Recipients. Am J Transplant (2014) 14:1912–21. 10.1111/ajt.12746 24954576

[11] Grigoryev DN Cheranova DI Heruth DP Huang P Zhang LQ Rabb H. Meta-Analysis of Molecular Response of Kidney to Ischemia Reperfusion Injury for the Identification of New Candidate Genes. BMC Nephrol (2013) 14:231. 10.1186/1471-2369-14-231 24152794 PMC4016589

[12] Damman J Bloks VW Daha MR van der Most PJ Sanjabi B van der Vlies P. Hypoxia and Complement-and-Coagulation Pathways in the Deceased Organ Donor as the Major Target for Intervention to Improve Renal Allograft Outcome. Transplantation (2015) 99:1293–300. 10.1097/TP.0000000000000500 25427168

[13] O’Connell PJ Zhang W Menon MC Yi Z Schroppel B Gallon L. Biopsy Transcriptome Expression Profiling to Identify Kidney Transplants at Risk of Chronic Injury: A Multicentre, Prospective Study. Lancet (2016) 388:983–93. 10.1016/S0140-6736(16)30826-1 PMC5014570 27452608

[14] Famulski KS Reeve J de Freitas DG Kreepala C Chang J Halloran PF. Kidney Transplants With Progressing Chronic Diseases Express High Levels of Acute Kidney Injury Transcripts. Am J Transplant (2013) 13:634–44. 10.1111/ajt.12080 23356967

[15] Fleige S Pfaffl MW. RNA Integrity and the Effect on the Real-Time qRT-PCR Performance. Mol Aspects Med (2006) 27:126–39. 10.1016/j.mam.2005.12.003 16469371

[16] Mutch DM Berger A Mansourian R Rytz A Roberts MA. The Limit Fold Change Model: A Practical Approach for Selecting Differentially Expressed Genes From Microarray Data. BMC Bioinf (2002) 3:17. 10.1186/1471-2105-3-17 PMC117238 12095422

[17] Duncan WC Shaw JL Burgess S McDonald SE Critchley HO Horne AW. Ectopic Pregnancy as a Model to Identify Endometrial Genes and Signaling Pathways Important in Decidualization and Regulated by Local Trophoblast. PloS One (2011) 6:e23595. 10.1371/journal.pone.0023595 21858178 PMC3157392

[18] Summers DM Johnson RJ Allen J Fuggle SV Collett D Watson CJ. Analysis of Factors That Affect Outcome After Transplantation of Kidneys Donated After Cardiac Death in the UK: A Cohort Study. Lancet (2010) 376:1303–11. 10.1016/S0140-6736(10)60827-6 20727576

[19] Waller JR Murphy GJ Bicknell GR Toomey D Nicholson ML. Effects of the Combination of Rapamycin With Tacrolimus or Cyclosporin on Experimental Intimal Hyperplasia. Br J Surg (2002) 89:1390–5. 10.1046/j.1365-2168.2002.02271.x 12390379

[20] Grimm PC Nickerson P Gough J McKenna R Stern E Jeffery J. Computerized Image Analysis of Sirius Red-Stained Renal Allograft Biopsies as a Surrogate Marker to Predict Long-Term Allograft Function. J Am Soc Nephrol (2003) 14:1662–8. 10.1097/01.asn.0000066143.02832.5e 12761269

[21] Saunders RN Bicknell GR Nicholson ML. The Impact of Cyclosporine Dose Reduction With or Without the Addition of Rapamycin on Functional, Molecular, and Histological Markers of Chronic Allograft Nephropathy. Transplantation (2003) 75:772–80. 10.1097/00007890-200303270-00008 12660500

[22] Fang H Harris SC Su Z Chen M Qian F Shi L. Arraytrack: An FDA and Public Genomic Tool. Methods Mol Biol (2017) 1613:333–53. 10.1007/978-1-4939-7027-8_13 28849567

[23] Zhao Z Yang C Wang L Li L Zhao T Hu L. The Regulatory T Cell Effector Soluble Fibrinogen-Like Protein 2 Induces Tubular Epithelial Cell Apoptosis in Renal Transplantation. Exp Biol Med (Maywood) (2014) 239:193–201. 10.1177/1535370213514921 24414480

[24] Oberbauer R Rohrmoser M Regele H Muhlbacher F Mayer G. Apoptosis of Tubular Epithelial Cells in Donor Kidney Biopsies Predicts Early Renal Allograft Function. J Am Soc Nephrol (1999) 10:2006–13. 10.1681/ASN.V1092006 10477154

[25] Zhao Z Yang C Li L Zhao T Wang L Rong R. Increased Peripheral and Local Soluble FGL2 in the Recovery of Renal Ischemia Reperfusion Injury in a Porcine Kidney Auto-Transplantation Model. J Trans Med (2014) 12:53. 10.1186/1479-5876-12-53 PMC3936847 24559374

[26] Ajay AK Saikumar J Bijol V Vaidya VS. Heterozygosity for Fibrinogen Results in Efficient Resolution of Kidney Ischemia Reperfusion Injury. PloS One (2012) 7:e45628. 10.1371/journal.pone.0045628 23029147 PMC3446934

[27] Hernandez-Saavedra D Zhou H McCord JM. Anti-Inflammatory Properties of a Chimeric Recombinant Superoxide Dismutase: SOD2/3. BioMed Pharmacother (2005) 59:204–8. 10.1016/j.biopha.2005.03.001 15862716

[28] Lim JH Kim EN Kim MY Chung S Shin SJ Kim HW. Age-Associated Molecular Changes in the Kidney in Aged Mice. Oxid Med Cell Longev (2012) 2012:171383. 10.1155/2012/171383 23326623 PMC3544311

[29] Chang J Ma JZ Zeng Q Cechova S Gantz A Nievergelt C. Loss of GSTM1, A NRF2 Target, Is Associated With Accelerated Progression of Hypertensive Kidney Disease in the African American Study of Kidney Disease (Aask). Am J Physiol Renal Physiol (2013) 304:F348–55. 10.1152/ajprenal.00568.2012 PMC3566499 23220723

[30] Singh R Manchanda PK Kesarwani P Srivastava A Mittal RD. Influence of Genetic Polymorphisms in GSTM1, Gstm3, GSTT1 and GSTP1 on Allograft Outcome in Renal Transplant Recipients. Clin Transplant (2009) 23:490–8. 10.1111/j.1399-0012.2009.00985.x 19486347

[31] Chang HR Tsai JP Yang SF Lin CK Lian JD. Glutathione S-transferase M1 Gene Polymorphism Is Associated With Susceptibility to Impaired Long-Term Allograft Outcomes in Renal Transplant Recipients. World J Surg (2013) 37:466–72. 10.1007/s00268-012-1815-6 23073505

[32] Lannan EA Galliher-Beckley AJ Scoltock AB Cidlowski JA. Proinflammatory Actions of Glucocorticoids: Glucocorticoids and TNFalpha Coregulate Gene Expression *In Vitro* and *In Vivo.* Endocrinology (2012) 153:3701–12. 10.1210/en.2012-1020 PMC3404340 22673229

[33] Asakura M Kitakaze M. Global Gene Expression Profiling in the Failing Myocardium. Circ J (2009) 73:1568–76. 10.1253/circj.cj-09-0465 19638707

[34] Takahashi E Okumura A Unoki-Kubota H Hirano H Kasuga M Kaburagi Y. Differential Proteome Analysis of Serum Proteins Associated With the Development of Type 2 Diabetes Mellitus in the KK-A(y) Mouse Model Using the iTRAQ Technique. J Proteomics (2013) 84:40–51. 10.1016/j.jprot.2013.03.014 23545169

[35] Turnier JL Brunner HI Bennett M Aleed A Gulati G Haffey WD. Discovery of SERPINA3 as a Candidate Urinary Biomarker of Lupus Nephritis Activity. Rheumatol (Oxf) (2019) 58:321–30. 10.1093/rheumatology/key301 PMC6343468 30285245

[36] Sanchez-Navarro A Mejia-Vilet JM Perez-Villalva R Carrillo-Perez DL Marquina-Castillo B Gamba G. SerpinA3 in the Early Recognition of Acute Kidney Injury to Chronic Kidney Disease (CKD) Transition in the Rat and its Potentiality in the Recognition of Patients With CKD. Sci Rep (2019) 9:10350. 10.1038/s41598-019-46601-1 31316093 PMC6637202

[37] Sano C Shimizu T Sato K Kawauchi H Tomioka H. Effects of Secretory Leucocyte Protease Inhibitor on the Production of the Anti-Inflammatory Cytokines, IL-10 and Transforming Growth Factor-Beta (TGF-Beta), by Lipopolysaccharide-Stimulated Macrophages. Clin Exp Immunol (2000) 121:77–85. 10.1046/j.1365-2249.2000.01269.x 10886242 PMC1905674

[38] Maruyama M Hay JG Yoshimura K Chu CS Crystal RG. Modulation of Secretory Leukoprotease Inhibitor Gene Expression in Human Bronchial Epithelial Cells by Phorbol Ester. J Clin Invest (1994) 94:368–75. 10.1172/JCI117331 PMC296318 7913712

[39] Bergenfeldt M Bjork P Ohlsson K. The Elimination of Secretory Leukocyte Protease Inhibitor (SLPI) After Intravenous Injection in Dog and Man. Scand J Clin Lab Invest (1990) 50:729–37. 10.1080/00365519009091066 2293334

[40] Ohlsson S Ljungkrantz I Ohlsson K Segelmark M Wieslander J. Novel Distribution of the Secretory Leucocyte Proteinase Inhibitor in Kidney. Mediators Inflamm (2001) 10:347–50. 10.1080/09629350120102389 PMC1781734 11817677

[41] Wilflingseder J Sunzenauer J Toronyi E Heinzel A Kainz A Mayer B. Molecular Pathogenesis of Post-Transplant Acute Kidney Injury: Assessment of Whole-Genome mRNA and miRNA Profiles. PloS One (2014) 9:e104164. 10.1371/journal.pone.0104164 25093671 PMC4122455

[42] Averdunk L Ruckbeil MV Zarbock A Martin L Marx G Jalaie H. SLPI - A Biomarker of Acute Kidney Injury After Open and Endovascular Thoracoabdominal Aortic Aneurysm (Taaa) Repair. Sci Rep (2020) 10:3453. 10.1038/s41598-020-60482-9 32103084 PMC7044192

[43] Averdunk L Fitzner C Levkovich T Leaf DE Sobotta M Vieten J. Secretory Leukocyte Protease Inhibitor (Slpi)-A Novel Predictive Biomarker of Acute Kidney Injury After Cardiac Surgery: A Prospective Observational Study. J Clin Med (2019) 8:1931. 10.3390/jcm8111931 PMC6912354 31717603

[44] Yan Q Sui W Wang B Zou H Zou G Luo H. Expression of MMP-2 and TIMP-1 in Renal Tissue of Patients With Chronic Active Antibody-Mediated Renal Graft Rejection. Diagn Pathol (2012) 7:141. 10.1186/1746-1596-7-141 23057632 PMC3539883

[45] Hirt-Minkowski P Marti HP Honger G Grandgirard D Leib SL Amico P. Correlation of Serum and Urinary Matrix Metalloproteases/Tissue Inhibitors of Metalloproteases With Subclinical Allograft Fibrosis in Renal Transplantation. Transpl Immunol (2014) 30:1–6. 10.1016/j.trim.2013.11.004 24291496

[46] Zawada AM Rogacev KS Muller S Rotter B Winter P Fliser D. Massive Analysis of Cdna Ends (MACE) and miRNA Expression Profiling Identifies Proatherogenic Pathways in Chronic Kidney Disease. Epigenetics (2014) 9:161–72. 10.4161/epi.26931 PMC3928179 24184689

[47] Mutoh J Ohsawa M Hisa H. Effect of Naloxone on Ischemic Acute Kidney Injury in the Mouse. Neuropharmacology (2013) 71:10–8. 10.1016/j.neuropharm.2013.03.001 23523991

[48] Catar R Witowski J Wagner P Annett Schramm I Kawka E Philippe A. The Proto-Oncogene c-Fos Transcriptionally Regulates VEGF Production During Peritoneal Inflammation. Kidney Int (2013) 84:1119–28. 10.1038/ki.2013.217 23760290

[49] Ishida M Ueki M Morishita J Ueno M Shiozawa S. Maekawa N. T-5224, a Selective Inhibitor of c-Fos/activator Protein-1, Improves Survival by Inhibiting Serum High Mobility Group Box-1 in Lethal Lipopolysaccharide-Induced Acute Kidney Injury Model. J Intensive Care (2015) 3:49. 10.1186/s40560-015-0115-2 26579229 PMC4647501

[50] Summers KM Bush SJ Hume DA. Network Analysis of Transcriptomic Diversity Amongst Resident Tissue Macrophages and Dendritic Cells in the Mouse Mononuclear Phagocyte System. PloS Biol (2020) 18:e3000859. 10.1371/journal.pbio.3000859 33031383 PMC7575120

[51] Kitamura M Ishikawa Y Moreno-Manzano V Xu Q Konta T Lucio-Cazana J. Intervention by Retinoic Acid in Oxidative Stress-Induced Apoptosis. Nephrol Dialysis Transplant: Off Publ Eur Dialysis Transplant Assoc - Eur Renal Assoc (2002) 17(Suppl 9):84–7. 10.1093/ndt/17.suppl_9.84 12386300

[52] Lin H Cheng CF. Activating Transcription Factor 3, an Early Cellular Adaptive Responder in Ischemia/Reperfusion-Induced Injury. Ci Ji Yi Xue Za Zhi (2018) 30:61–5. 10.4103/tcmj.tcmj_37_18 PMC5968744 29875584

[53] Cohen DM. Urea-Inducible Egr-1 Transcription in Renal Inner Medullary Collecting Duct (mIMCD3) Cells is Mediated by Extracellular Signal-Regulated Kinase Activation. Proc Natl Acad Sci USA (1996) 93:11242–7. 10.1073/pnas.93.20.11242 PMC38314 8855340

[54] Mao Y Vyas NK Vyas MN Chen DH Ludtke SJ Chiu W. Structure of the Bifunctional and Golgi-Associated Formiminotransferase Cyclodeaminase Octamer. EMBO J (2004) 23:2963–71. 10.1038/sj.emboj.7600327 PMC514939 15272307

[55] Hennig D Scales SJ Moreau A Murley LL De Mey J Kreis TE. A Formiminotransferase Cyclodeaminase Isoform Is Localized to the Golgi Complex and Can Mediate Interaction of Trans-Golgi Network-Derived Vesicles With Microtubules. J Biol Chem (1998) 273:19602–11. 10.1074/jbc.273.31.19602 9677386

[56] Gao YS Vrielink A MacKenzie R Sztul E. A Novel Type of Regulation of the Vimentin Intermediate Filament Cytoskeleton by a Golgi Protein. Eur J Cell Biol (2002) 81:391–401. 10.1078/0171-9335-00260 12160147

[57] Charrin S Jouannet S Boucheix C Rubinstein E. Tetraspanins at a Glance. J Cell science (2014) 127:3641–8. 10.1242/jcs.154906 25128561

[58] Arcaroli JJ Touban BM Tan AC Varella-Garcia M Powell RW Eckhardt SG. Gene Array and Fluorescence *In Situ* Hybridization Biomarkers of Activity of Saracatinib (AZD0530), A Src Inhibitor, in a Preclinical Model of Colorectal Cancer. Clin Cancer Res (2010) 16:4165–77. 10.1158/1078-0432.CCR-10-0066 PMC3805460 20682712

[59] Cheong CM Chow AW Fitter S Hewett DR Martin SK Williams SA. Tetraspanin 7 (TSPAN7) Expression Is Upregulated in Multiple Myeloma Patients and Inhibits Myeloma Tumour Development *In Vivo* . Exp Cell Res (2015) 332:24–38. 10.1016/j.yexcr.2015.01.006 25637218

[60] Wuttig D Zastrow S Fussel S Toma MI Meinhardt M Kalman K. Cd31, EDNRB and TSPAN7 Are Promising Prognostic Markers in Clear-Cell Renal Cell Carcinoma Revealed by Genome-Wide Expression Analyses of Primary Tumors and Metastases. Int J Cancer (2012) 131:E693–704. 10.1002/ijc.27419 22213152

[61] Waters AM Licht C. aHUS Caused by Complement Dysregulation: New Therapies on the Horizon. Pediatr Nephrol (2011) 26:41–57. 10.1007/s00467-010-1556-4 20556434 PMC2991208

[62] Le Quintrec M Lionet A Kamar N Karras A Barbier S Buchler M. Complement Mutation-Associated *De Novo* Thrombotic Microangiopathy Following Kidney Transplantation. Am J Transplant (2008) 8:1694–701. 10.1111/j.1600-6143.2008.02297.x 18557729

[63] Le Quintrec M Zuber J Moulin B Kamar N Jablonski M Lionet A. Complement Genes Strongly Predict Recurrence and Graft Outcome in Adult Renal Transplant Recipients With Atypical Hemolytic and Uremic Syndrome. Am J Transplant (2013) 13:663–75. 10.1111/ajt.12077 23356914

[64] Akalin E Hendrix RC Polavarapu RG Pearson TC Neylan JF Larsen CP. Gene Expression Analysis in Human Renal Allograft Biopsy Samples Using High-Density Oligoarray Technology. Transplantation (2001) 72:948–53. 10.1097/00007890-200109150-00034 11571464

[65] Scherer A Krause A Walker JR Korn A Niese D Raulf F. Early Prognosis of the Development of Renal Chronic Allograft Rejection by Gene Expression Profiling of Human Protocol Biopsies. Transplantation (2003) 75:1323–30. 10.1097/01.TP.0000068481.98801.10 12717224

[66] Yuen PS Jo SK Holly MK Hu X Star RA. Ischemic and Nephrotoxic Acute Renal Failure are Distinguished by Their Broad Transcriptomic Responses. Physiol Genomics (2006) 25:375–86. 10.1152/physiolgenomics.00223.2005 PMC1502395 16507785

[67] Berglund SR Schwietert CW Jones AA Stern RL Lehmann J Goldberg Z. Optimized Methodology for Sequential Extraction of RNA and Protein From Small Human Skin Biopsies. J Invest Dermatol (2007) 127:349–53. 10.1038/sj.jid.5700557 17039245

[68] Halloran PF Reeve J Akalin E Aubert O Bohmig GA Brennan D. Real Time Central Assessment of Kidney Transplant Indication Biopsies by Microarrays: The INTERCOMEX Study. Am J Transplant (2017) 17:2851–62. 10.1111/ajt.14329 28449409

[69] Yang B Elias JE Bloxham M Nicholson ML. Synthetic Small Interfering RNA Down-Regulates Caspase-3 and Affects Apoptosis, IL-1 Beta, and Viability of Porcine Proximal Tubular Cells. J Cell Biochem (2011) 112:1337–47. 10.1002/jcb.23050 21321992

[70] Wu Y Chen W Zhang Y Liu A Yang C Wang H. Potent Therapy and Transcriptional Profile of Combined Erythropoietin-Derived Peptide Cyclic Helix B Surface Peptide and Caspase-3 siRNA Against Kidney Ischemia/Reperfusion Injury in Mice. J Pharmacol Exp Ther (2020) 375:92–103. 10.1124/jpet.120.000092 32759272

[71] Yang B Hosgood SA Nicholson ML. Naked Small Interfering RNA of Caspase-3 in Preservation Solution and Autologous Blood Perfusate Protects Isolated Ischemic Porcine Kidneys. Transplantation (2011) 91:501–7. 10.1097/TP.0b013e318207949f 21192320

[72] Yang C Zhao T Zhao Z Jia Y Li L Zhang Y. Serum-Stabilized Naked Caspase-3 siRNA Protects Autotransplant Kidneys in a Porcine Model. Mol Ther (2014) 22:1817–28. 10.1038/mt.2014.111 PMC4428396 24930602

[73] Zwaini Z Patel M Stover C Dormer J Nicholson ML Hosgood SA. Comparative Analysis of Risk Factors in Declined Kidneys From Donation After Brain Death and Circulatory Death. Medicina (Kaunas) (2020) 56:313–25. 10.3390/medicina56060317 PMC7353903 32604873

