# Supplementary material for: Identifying Biomarkers from Transcriptomic Signatures in Renal Allograft Biopsies Using Deceased and Living Donors
Source: Front Immunol. 2021 Jul 1;12:657860. doi: 10.3389/fimmu.2021.657860 (PMC8282197; doi:10.3389/fimmu.2021.657860)
Supplement: Supplementary Table 2 — 25 DEGs (No.1-25) between DD & LD in common at 30 min and 3 months, and 10 DEGs (No.2, 3, 19 and 26-32) at 30 minutes correlated with both SCr and SR staining for further qPCR detection to identify biomarkers. SCr, serum creatinine; SR, Sirius red staining. [file DataSheet_2.docx]

**Supplemental Table 2.**
