# Supplementary material for: Identifying Biomarkers from Transcriptomic Signatures in Renal Allograft Biopsies Using Deceased and Living Donors
Source: Front Immunol. 2021 Jul 1;12:657860. doi: 10.3389/fimmu.2021.657860 (PMC8282197; doi:10.3389/fimmu.2021.657860)
Supplement: Supplementary Table 3 — DEGs revealed by microarray analysis and associated with kidney injury or allograft rejection from this study and previous publications. There were some commonly DEGs (underline) between studies. [file DataSheet_3.docx]

**Supplemental Table 3.**
